# Fine Root Abundance and Dynamics of Stone Pine (*Pinus cembra*) at the Alpine Treeline Is Not Impaired by Self-shading

**DOI:** 10.3389/fpls.2017.00602

**Published:** 2017-04-19

**Authors:** Petra Kubisch, Christoph Leuschner, Heinz Coners, Andreas Gruber, Dietrich Hertel

**Affiliations:** ^1^Plant Ecology and Ecosystems Research, Albrecht-von-Haller Institute for Plant Sciences, University of GöttingenGöttingen, Germany; ^2^Research Group Dendroecology and Tree Physiology, Institute of Botany, University of InnsbruckInnsbruck, Austria

**Keywords:** Austrian Alps, fine root biomass, fine root mortality, fine root morphology, fine root turnover, soil temperature

## Abstract

Low temperatures are crucial for the formation of the alpine treeline worldwide. Since soil temperature in the shade of tree canopies is lower than in open sites, it was assumed that self-shading may impair the trees’ root growth performance. While experiments with tree saplings demonstrate root growth impairment at soil temperatures below 5–7°C, field studies exploring the soil temperature – root growth relationship at the treeline are missing. We recorded soil temperature and fine root abundance and dynamics in shaded and sun-exposed areas under canopies of isolated *Pinus cembra* trees at the alpine treeline. In contrast to the mentioned assumption, we found more fine root biomass and higher fine root growth in colder than in warmer soil areas. Moreover, colder areas showed higher fine root turnover and thus lower root lifespan than warmer places. We conclude that *P. cembra* balances enhanced fine root mortality in cold soils with higher fine root activity and by maintaining higher fine root biomass, most likely as a response to shortage in soil resource supply. The results from our study highlight the importance of *in situ* measurements on mature trees to understand the fine root response and carbon allocation pattern to the thermal growth conditions at the alpine treeline.

## Introduction

The alpine treeline is one of the most conspicuous vegetation boundaries on earth, which has been studied by ecologists and geographers for decades. However, the causes for the formation of this steep ecotone are still intensively debated. The majority of scientists concerned with the topic may agree that adverse thermal conditions at (and above) the alpine treeline ecotone are crucial for explaining treeline formation. While a ‘classic’ explanation assumes that insufficient photosynthetic carbon gain is limiting tree growth at the treeline (‘carbon-source limitation hypothesis’; see reviews by Troll, 1973; Tranquillini, 1979; Stevens and Fox, 1991; Körner, 1998, 2012a; Holtmeier, 2009), it has been proposed more recently that cold temperatures directly are limiting metabolic activity and hence cell division and cell growth in meristematic tissues (referred to as ‘carbon-sink limitation hypothesis’; Körner, 1998, 2012a; see also Hoch et al., 2002; Hoch and Körner, 2003). Microclimatic measurements at various treelines indicate that the elevational position of the alpine treeline apparently is more closely related to soil than to air temperature (e.g., Sveinbjörnsson, 2000; Körner and Hoch, 2006; Körner, 2012a). In a global survey of soil temperature data from treeline habitats, Körner and Paulsen (2004) found the treeline position to coincide with a 6.7°C growing season mean in 10 cm soil depth. According to Körner (2012a), this correlation does not necessarily show a more prominent soil temperature than air temperature effect on tree growth, but may simply reflect lower diurnal and seasonal variation in soil than air temperature at the treeline (Körner and Paulsen, 2004). Moreover, the apparently critical temperature of ∼6.7°C experienced by treeline trees may not directly relate to a low temperature threshold of biological processes at this temperature (Körner, 1998, 2012a).

For most processes of carbon turnover, the temperature dependence of metabolic activity in treeline trees is not precisely known. Where empirical data are available, they often originate from sapling studies in greenhouses, such as the experiments on low-temperature limits of root growth in temperate tree species conducted by Alvarez-Uria and Körner (2007), Hoch and Körner (2009), Körner (2012b), and Schenker et al. (2014). Accordingly, root growth of tree saplings is greatly reduced below ∼5°C, which may represent a signal to slow down shoot growth (see literature review in Körner, 2003). These findings were applied to the treeline question by postulating that self-shading by a closed canopy at the treeline impairs aboveground and belowground growth of trees more than under single tree individuals above the treeline, where more light penetrates to the ground and soil temperatures may be more favorable (Körner, 1998, 2012b; Li et al., 2003). The self-shading hypothesis predicts that the upper limit of closed forest is partly a consequence of an unfavorable soil thermal regime caused by the trees themselves. However, convincing evidence from field studies in support of this hypothesis does not yet exist.

In apparent contrast to the experimental results on root growth impairment at low temperatures in tree saplings, findings from field studies in mature forests along elevation transects show a pronounced increase in fine root biomass with decreasing temperature toward the alpine treeline (Kitayama and Aiba, 2002; Leuschner et al., 2007; Hertel and Wesche, 2008; Hertel et al., 2008; Hertel and Schöling, 2011a). Furthermore, several authors detected no root productivity differences, or even higher fine root growth rates, at the treeline compared to stands at lower elevation (Graefe et al., 2008; Hertel and Schöling, 2011b; Mao et al., 2013). This apparent contradiction calls for a detailed study of root zone temperatures and tree root growth at the alpine treeline.

We examined the temperature dependence of fine root biomass and fine root productivity in isolated Stone pine trees (*Pinus cembra* L.) in the alpine treeline ecotone. We synchronously mapped soil temperature and fine root biomass at high resolution in six plots and compared fine root productivity and turnover between sunny and shaded soil patches under the canopy of 12 pine trees. The study aim was to search for evidence in support of the postulated self-shading effect of tree canopies on the soil thermal regime and related negative effects on soil biomass and productivity in the treeline ecotone. We conducted root coring for fine root biomass at 216 locations with variable irradiance and soil thermal conditions and measured root productivity and fine root turnover with the ingrowth core technique at 24 sunny or shaded locations.

With reference to the assumptions made by Körner (1998, 2012b), we tested the hypotheses that (a) tree fine root biomass in sunny and shaded patches of the treeline ecotone is more closely related to the soil thermal regime developed in the afternoon than in the morning hours; (b) tree fine root biomass density is lower in shaded, cooler patches under the canopy than in sunny, warmer areas; and (c) tree fine root productivity is considerably lower in shaded, cooler patches under the canopy than in sunny, warmer areas. The study was conducted in mid-summer, when thermal differences between sunny and shady areas in the treeline ecotone were greatest.

## Materials and Methods

### Study Site

**FIGURE 1:**
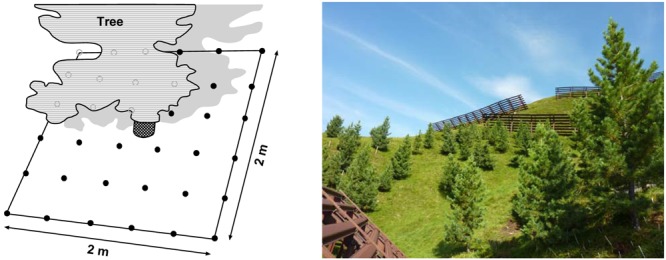
**Left: 4 m^2^ plot with 40 cm × 40 cm-grid scheme with the 36 sampling points used for temperature measurement and root sampling with the *Pinus cembra* tree close to the plot center.** Due to their position on a south-facing slope, the plots were shade by the tree in their upper part while being sun-exposed in their lower part. Right: image of the upper part of the afforestation area in the Austrian Alps containing the study area (photo credit: P. Kubisch).

The study was conducted in the treeline ecotone of the central Eastern Alps (Stubai Alps, Tyrol, Austria) with isolated Stone pine trees (*P. cembra* L.) representing the uppermost occurrence of trees. The study site belongs to a large former afforestation area on the ‘Haggener Sonnberg’ (47°12′42″ N, 11°5′04 ″ E) north of the upper Sellrain valley near St. Sigmund on a south-facing slope (25°) at ca. 2025 m a.s.l.. In the 1970s, this area was afforested with native Stone pine to provide protection against avalanches. It started as a pilot project at lower elevation and was continued in 1980 up to the potential treeline elevation, where the study site is located. Prior to afforestation, the south-facing slopes had been clear-cut and used for grazing by cattle and sheep for centuries. The pine trees in our study therefore all had roughly the same age (ca. 30 years) and similar aboveground stature. The soil of the study site is an oligotrophic podzolic leptosol (WRB classification) originating from gneiss and micaschist bedrock (Kronfuss and Havranek, 1999). The ca. 5 cm deep sandy-loamy mineral soil is covered by a humus layer of ca. 5 cm thickness (Wieser et al., 2015). The soil was completely covered by a dense layer of herbaceous vegetation together with some small shrub individuals (see image in **Figure 1**). Various soil chemical properties from the plots used for studying fine root growth are given in **Table 1**.

**Table 1:** Soil chemical properties from the upper 10 cm of the soil of the plots used for analyzing fine root growth and turnover in the vicinity of isolated *Pinus cembra* trees: given are minimum and maximum pH values (measured in water or in KCl), total nitrogen concentration, soil organic carbon (SOC), C/N ratio, resin-extractable phosphorus, cation exchange capacity (CEC), and base saturation.

pH (H_2_O/KCl)	Nitrogen (mg g^-1^)	SOC (mg g^-1^)	C_org_/N_org_ (g g^-1^)	P_resin_ (μmol g^-1^)	CEC (μmol_c_ g^-1^)	Base saturation (%)
4.1–4.8/3.5–3.9	8.7 ± 0.7	172.1 ± 16.6	19.8 ± 0.8	0.45 ± 0.04	188.1 ± 26.6	33.3 ± 6.2

*The soil samples origin from shaded and unshaded locations. However, we pooled the data since none of the variables were significantly different between the two location types.*

Mean annual air temperature at a nearby weather station at 1800 m a.s.l. (Kronfuss, 1997) was 3.2°C in the years 1975–1994 with February being the coldest month (mean temperature -3.5°C) and July the warmest (10.7°C). The mean annual precipitation is 909 mm with most precipitation occurring from May to October (610 mm). During the growing season (May to October) in the study year 2012, daily mean air temperature averaged 9.2°C and varied between -4.2°C on May 16th and 18.4 °C on August 20th; during this period, 889 mm of precipitation was recorded (Wieser et al., 2015).

### Study Design and Soil Temperature Measurements

For our investigation, we selected six Stone pine tree individuals which were representative for the whole afforestation site in terms of aboveground stature, slope position, and soil conditions. The six trees were between 2.8 (plot 2) and 4.2 m (plot 3) high with a stem diameter at breast height (130 cm) of 5.5–7.5 cm (**Table 2**); the tree individuals had a mean distance of ca. 10–20 m to each other. For each tree, we established a measuring and sampling plot of 2 m × 2 m size with the stem in the center of the plot. Each plot contained a systematic grid of 6 × 6 = 36 points (with 40 cm distance between two points) where soil temperature measurements and soil sampling for the quantitative analysis of living and dead *Pinus* fine roots were conducted (**Figure 1**).

**Table 2:** Mean diameter at breast height (dbh) and maximum height of the *P. cembra* trees as well as exposition (aspect) of the six study plots.

	Plot 1	Plot 2	Plot 3	Plot 4	Plot 5	Plot 6
Dbh (cm)	7	5.5	7	7.5	6	6
Tree height (m)	3.6	2.8	4.2	3.3	3.2	3.0

*All plots were south exposed.*

A spatially detailed mapping of soil temperatures under the canopy of the six *Pinus* tree plots was conducted on August 1, 2012, a completely cloudless day in the summer period of the year, when most pronounced thermal differences between sunny and shaded soil areas can be expected. Soil temperature was measured at 10 cm depth with a mobile thermometer at the each 36 grid points of the six study plots. Measurements were conducted during three periods of the day: The first measurement campaign was conducted at dawn (7:26 solar time at the first plot until 8:48 at the sixth plot) reflecting the soil conditions after maximum night cooling; the second campaign took place at noon (11:14 solar time in the first plot until 12:33 in the sixth plot), i.e., the period of highest solar radiation input, and the third campaign was conducted in the early afternoon between 14:42 solar time at the first plot until 16:00 in the sixth plot, representing the period of the daily maximum thermal differences in 10 cm soil depth through surface warming. All measurements were taken manually with the same soil thermometer (DET3R penetration thermometer, Voltcraft, Conrad Electronic AG, Wollerau, Switzerland) at each grid point in ascending plot order (starting with plot 1 and continuing to plot 6) until a constant temperature reading was achieved at the respective location.

A continuous long-term recording of soil temperature in 10 cm depth in shaded area and sunny areas of *Pinus* individuals was done under 12 trees in the vicinity of the six plots mentioned above that were selected to investigate fine root dynamics (see below). There were no significant differences in soil chemical or physical properties found between the shaded and sunny locations under the study trees.

### Analysis of Living and Dead *Pinus* Fine Root Mass

Soil samples for root abundance analysis were taken in 0–10 cm depth (which at most places represent the entire soil profile at these sites) at the same grid points on the day after measuring soil temperature using a steel corer of 35 mm in diameter. The soil samples were stored in plastic bags at 4°C until further processing within 6 weeks. The fine roots (roots <2 mm in diameter) were washed from the soil by rinsing the soil sample over a sieve with 0.25 mm mesh size. All fine roots longer than 10 mm were picked out with a pair of tweezers and root segments of *P. cembra* were separated under the stereomicroscope from other species (e.g., *Calluna vulgaris, Rhododendron ferrugineum, Arctostaphylos uva-ursi*, and herbaceous species) based on morphological characteristics. In this study, only *Pinus* fine roots were considered. The fine roots were sorted into biomass (living) and necromass (dead) under a stereomicroscope (6–40× magnification) using the criteria root turgescence, the constitution of periderm and stele as well as elasticity of the stele (Hertel and Leuschner, 2002; Hertel et al., 2013). All living fine roots of *Pinus* were scanned under water using an EPSON expression 1680 scanner (EPSON, Seiko Epson Coop., Owa, Japan), and a morphological analysis was conducted using WinRhizo 2005c software (Regent Instruments Inc., Ville de Québec, QC, Canada) to determine average fine root diameter, fine root length, surface area and root tissue density (RTD). Living and dead fine root mass was weighed after drying the material at 70°C for 48 h to constant weight. For a subsample of living root strands from sampling points that were assigned to the 25% coldest or 25% warmest locations in the plots, the number of root tips and their ectomycorrhizal colonization status were analyzed under the stereomicroscope. However, ectomycorrhizal species identity could not be analyzed in our study.

### Analysis of Annual Fine Root Production and Turnover in Their Dependence on Soil Temperature

For measuring annual fine root productivity in the study area, we selected 12 *P. cembra* trees adjacent to the six grid-sampling plots described above and installed one ingrowth core each in sunny patches (ca. 40 cm south of the tree stem) and shaded patches (ca. 40 cm north of the same stem), i.e., 24 ingrowth cores in total. In comparison with other techniques, the ingrowth core approach has been found to deliver relatively conservative fine root production estimates in temperate forests (e.g., Hertel and Leuschner, 2002; Hendricks et al., 2006; Finér et al., 2011a). Cores of 35 mm diameter and 10 cm length (which covered the mineral topsoil and the organic layer) were installed in April 2011 and re-sampled after 24 months in April 2013. For installing the ingrowth cores, the upper 10 cm of the soil was extracted with the soil corer. Bulk soil of the same soil depth from adjacent places was cleaned by hand from all macroscopically visible live and dead rootlets in the field, and the soil material was subsequently refilled into the hole by conserving the natural sequence of horizons. Care was taken to maintain the natural structure and density of the soil as accurately as possible; no mesh gaze was used to guarantee a full contact of the refilled soil to the surrounding natural soil material. The exact position of each ingrowth core was marked with three plastic sticks to allow re-sampling. Field studies in temperate forests have shown that tree fine roots typically start recolonizing root-free soil after a time lag of about 1 year (Hertel and Leuschner, 2002; Meinen et al., 2009; Hertel et al., 2013). We therefore assumed that the ingrowth period started in spring 2012. In April 2013, the cores were re-sampled, taken to the laboratory and processed as described above. Annual fine root production (in g m^-2^ yr^-1^) was estimated by relating the total mass of living and dead *Pinus* roots in the ingrowth core samples to 1 year and to one square meter ground area.

To calculate fine root turnover (and its inverse, mean fine root lifespan), we analyzed the mass of living and dead *Pinus* fine roots in the soil samples that were initially extracted from the ingrowth core holes, applying the same procedure as described above. Fine root turnover was calculated by dividing annual fine root production in the ingrowth cores by the initial fine root biomass at the ingrowth core locations.

To compare the soil temperature of the sunny and shaded ingrowth core locations (sunny: ca. 40 cm south of the tree stem, i.e., downslope; shaded: ca. 40 cm north of the same tree stem, i.e., upslope), miniature temperature sensors and data loggers (‘iButtons,’ Maxim Integrated, San Jose, CA, USA) were installed in direct vicinity of each ingrowth core in 10 cm soil depth to record soil temperature continuously in bi-hourly intervals over the whole ingrowth period.

### Statistical Analyses

All variable samples were tested for normal distribution using a Shapiro–Wilk test. Since in most cases normal distribution was not given, we applied a one-way Kruskal–Wallis single factor analysis of variance and, subsequently, a non-parametric Mann–Whitney two-sample test (*U* test) to locate significant differences. A significance level of *p* < 0.05 was used in all statistical tests. To analyze the shading effect under the isolated *Pinus* trees on soil temperature and fine root abundance in the six plots, we divided the 36 grid points in each plot into four temperature categories (#1 to #4) which represented the four quartiles (*n* = 9 temperature values per quartile and plot) of the 36 ranked temperature values measured in each plot; category #1 represented the 9 grid points with the lowest recorded temperature values, category #2 those with the 9 next-higher temperature values, category #3 the 9 points the second-highest values, and category #4 contained the 9 grid points with the highest soil temperatures.

The distribution of soil temperatures in the plots during the third (afternoon) measurement campaign was visualized in maps by interpolating between the data points using Xact8 software (SciLab, Hamburg, Germany). A regression analysis for the dependence of fine root biomass and necromass on soil temperature was conducted using the same software. Temperature effects on fine root biomass, and cumulative fine root length and area per unit soil volume were tested with a correlation analysis using the PROC CORR routine in SAS 9.3 software. In the correlation analyses, either absolute soil temperatures or standardized temperature values in form of the relative deviation of temperature values at each grid point from the temperature maximum recorded in a plot and measurement period were used. Relative soil temperature values were calculated in order to compare the thermal variation in the plots independently of absolute deviations in the temperature regime of the six plots.

## Results

### Soil Temperature Patterns under the *Pinus* Crowns

**FIGURE 2:**
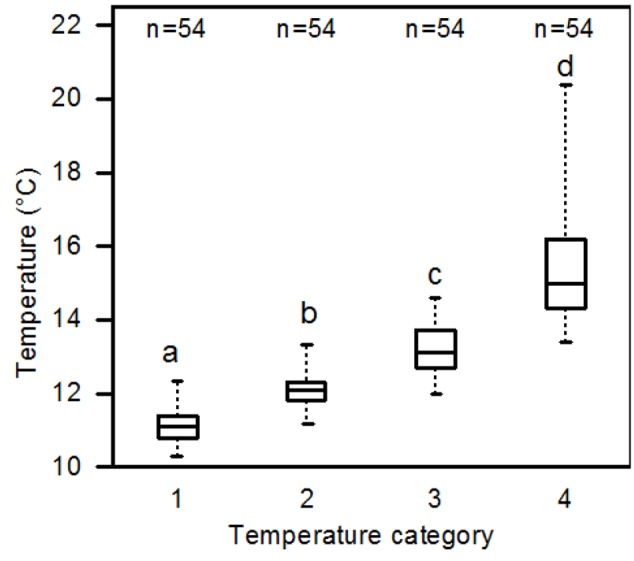
**Median soil temperatures recorded in the temperature categories #1 to #4 which represent the 4 quartiles of the 36 measuring points in a plot (#1: the 25% coldest temperature values, #4: the 25% warmest temperature values) (all six plots pooled; afternoon measurements taken on August 1, 2012, at 10 cm depth).** All four categories differed significantly form each other (*p* < 0.05; Mann–Whitney *U* test).

On the cloudless day August 1, 2012, temperature mapping showed a soil temperature average of 10.1°C for the dawn measuring period (07:26–08:48 solar time) and of 13.0°C for the afternoon period (14:42–16:00 solar time) in the six study plots (6 × 36 measuring points) around the *P. cembra* trees (**Table 3**). The recorded absolute maximum temperature was 20.4°C during the afternoon measurement, the absolute minimum temperature was 8.6°C during the dawn measurement. While the mean minimum soil temperature varied only slightly over the day in the shaded areas (9.0–10.6 °C), patches that were warmed by direct solar irradiance showed a much larger variability of mean maximum soil temperature (11.0–17.5°C; **Table 3**). Correspondingly, the diurnal variation in absolute minimum temperature was low (ranging between 8.6°C at dawn and 10.3°C in the afternoon), while the absolute maxima varied from 11.4°C (dawn) to 20.4°C (afternoon) (**Table 3** and Supplementary Figure SI1). Categorizing the measuring points by means of their median temperature into the four quartiles produced the temperature categories #1 to #4 which differed significantly from each other (**Figure 2**).

**Table 3:** Daily mean, and mean or absolute minima and maxima of soil temperature measured at 10 cm depth on a cloudless day in mid-summer (August 1, 2012) in the six plots at dawn, noon and in the afternoon (see text).

Time	Mean	Mean minimum	Mean maximum	Absolute minimum	Absolute maximum
Dawn	10.1 ± 0.1	9.0 ± 0.1	11.0 ± 0.2	8.6	11.4
Noon	11.0 ± 0.1	9.8 ± 0.1	13.3 ± 0.6	9.6	16.1
Afternoon	13.0 ± 0.2	10.6 ± 0.1	17.5 ± 1.0	10.3	20.4

*Given are means ± SE for all 36 grid points of the six study plots.*

**FIGURE 3:**
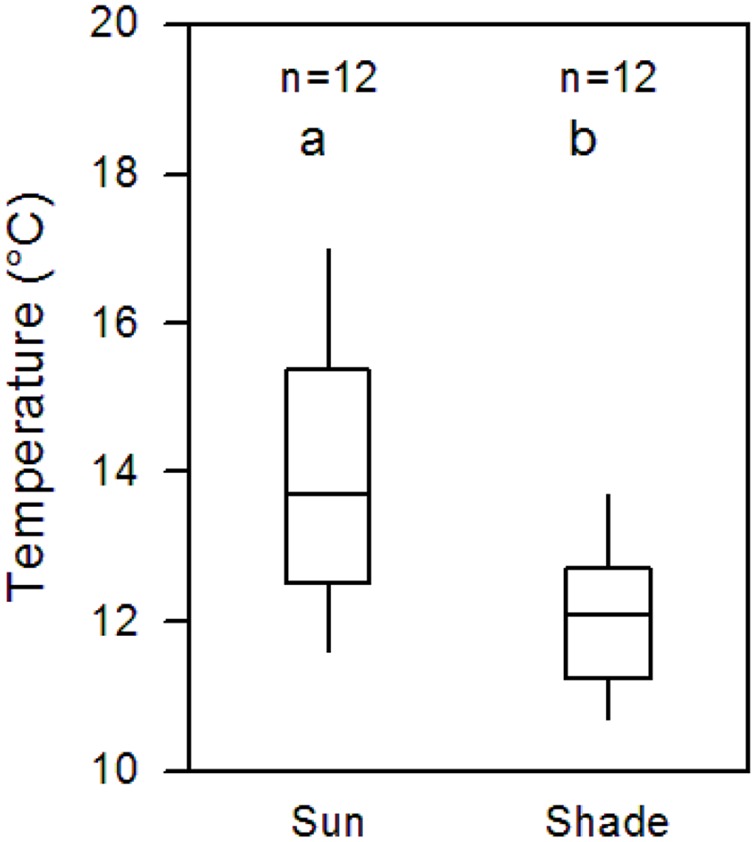
**Soil temperature difference between the shaded (north of the stem) and sun-exposed side (south of the stem) of the slope under 12 isolated *P. cembra* trees.** Given are the median and four quartiles of the afternoon temperatures in July and August 2012 (14:00–16:00 solar time) measured in 10 cm soil depth. Both groups are significantly different (*p* < 0.001; Mann–Whitney *U* test).

Continuous soil temperature measurement in 10 cm depth in July and August 2012 in the sunny and shaded patches adjacent to the 12 *Pinus* trees of the ingrowth core study revealed a by 1.8 Kelvin higher mean afternoon temperature in the sunny plots (difference significant; **Figure 3**). Even the difference of continuous whole-day soil temperature measurements during the entire growing season (May to September) was still 1.0 Kelvin between shaded (9.8°C) and sunny patches (10.8°C) although night, dawn, and evening temperatures were included.

### Fine Root Morphological Traits

For most traits, the fine root morphology of *P. cembra* did not differ between warmer and colder soil patches. Mean root diameter was almost identical in soil samples of the four temperature categories, while specific root length (SRL), specific root surface area (SRA), and RTD showed only moderate variation across this thermal gradient (**Table 4**). Similarly, *Pinus* root samples from the coldest (#1) and warmest temperature categories (#4) did not differ in the degree of ectomycorrhizal colonization of root tips (84% vs. 82%; 216 samples analyzed in total; data not shown). However, the fine roots in the coldest plots had more than twice the number of root tips per sample as compared to the roots of the warmest plots (categories #1 and #4: 24 and 11 tips per sample; *p* < 0.01).

**Table 4:** Mean diameter, specific root length (SRL), specific root surface area (SRA), and root tissue density (RTD) of fine roots collected at locations assigned to the four soil temperature categories (*n* = 6 plots); category #1 represents the 25% coldest locations, category #4 the 25% warmest locations.

Category	Mean diameter (mm)	SRL (m g^-1^)	SRA (cm^2^ g^-1^)	RTD (g cm^-3^)
1	0.612 ± 0.016	11.632 ± 0.328	210.4 ± 6.5	0.34 ± 0.022
2	0.635 ± 0.012	10.013 ± 0.853	187.9 ± 15.2	0.53 ± 0.169
3	0.629 ± 0.025	11.942 ± 1.008	211.1 ± 12.3	0.36 ± 0.021
4	0.645 ± 0.019	11.458 ± 0.874	212.1 ± 14.4	0.38 ± 0.049

*In total, 216 root samples were analyzed. None of the differences between the four temperature category means for the four morphological root traits were statistically significant at *P* < 0.05.*

### Standing Fine Root Biomass and Necromass

**FIGURE 4:**
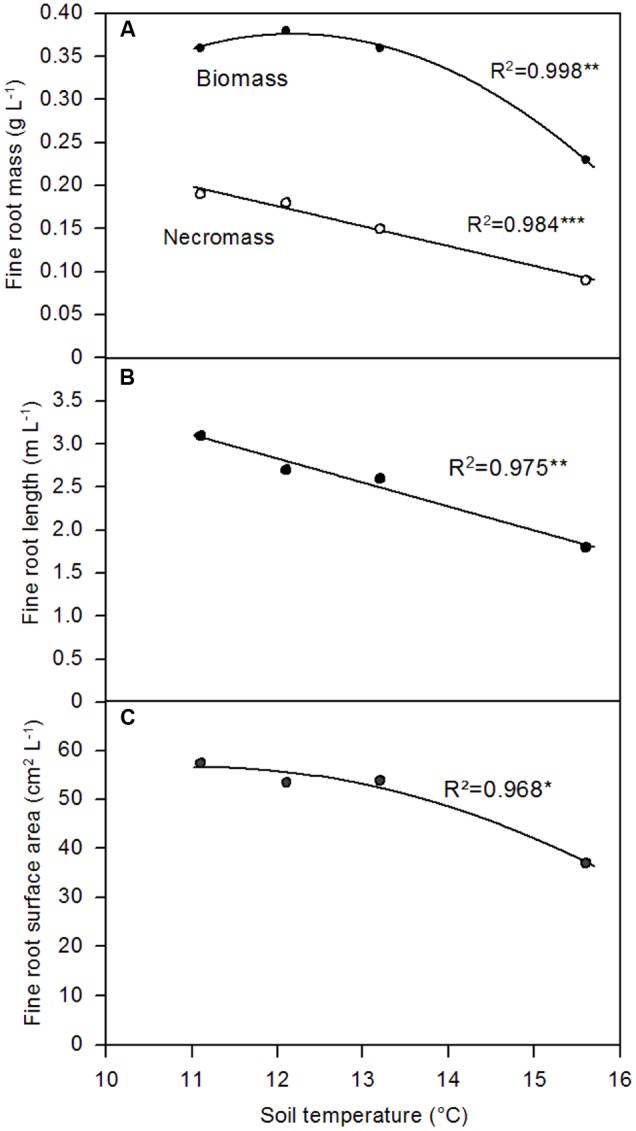
**Dependence of fine root biomass and necromass density (A)**, cumulative fine root length per soil volume **(B)**, and cumulative fine root surface area per volume **(C)** of *P. cembra* on median soil temperature measured at 10 cm depth. The measuring points in the plots were assigned to the four temperature categories #1 to #4 which represent the quartiles of soil temperature values measured in the six plots during the early afternoon measurement; ^∗^*p* < 0.05, ^∗∗^*p* < 0.01, ^∗∗∗^*p* < 0.001.

Fine root biomass (or cumulative fine root length and surface area) in the plots showed a significant negative relation to soil temperature (absolute values or deviation from the absolute maximum per plot) in case of the afternoon temperature measurement. This relationship was less clear for the noon temperature data (only significant for root length and area but not for biomass) and disappeared for the temperature measurements at dawn (**Table 5**). Similarly, when the plots were assigned to the four temperature classes, linear or non-linear negative relationships between fine root biomass (or cumulative fine root length or surface area) and median afternoon temperature in the temperature classes #1 to #4 were detected in the range from 11.0 to 15.5°C (**Figures 4A–C**). A similar relation existed for fine root necromass. These findings match the observation that both fine root biomass and necromass were significantly lower in the southeastern quarters of the plots with highest solar irradiance as compared to the northeastern and northwestern quarters, where shading by the tree canopy was most effective (data not shown).

**Table 5:** Correlation between absolute soil temperature or normalized temperature (i.e., relative temperature deviation at a grid point from the soil temperature maximum in a plot) per plot and measurement period and fine root biomass, cumlative fine root length and cumulative fine root surface area per soil volume during the dawn, noon, and afternoon measurements.

Time	Variable	Absolute temperature	Normalized temperature
		*r*	*r*
Dawn	Biomass (g L^-1^)	–0.003	–0.071
	Length (m L^-1^)	–0.029	–0.036
	Surface area (m^2^ L^-1^)	–0.031	–0.049
Noon	Biomass (g L^-1^)	–0.102	–0.109
	Length (m L^-1^)	–0.146^∗^	–0.194^∗∗^
	Surface area (m^2^ L^-1^)	–0.139^∗^	–0.206^∗∗^
Afternoon	Biomass (g L^-1^)	–0.164^∗^	–0.297^∗∗∗^
	Length (m L^-1^)	–0.178^∗∗^	–0.327^∗∗∗^
	Surface area (m^2^ L^-1^)	–0.159^∗^	–0.308^∗∗∗^

*Pearson correlation coefficients (*r*) and significance levels (^∗^*p* < 0.05; ^∗∗^*p* < 0.01; ^∗∗∗^*p* < 0.001) are shown.*

### Fine Root Growth and Turnover

**FIGURE 5:**
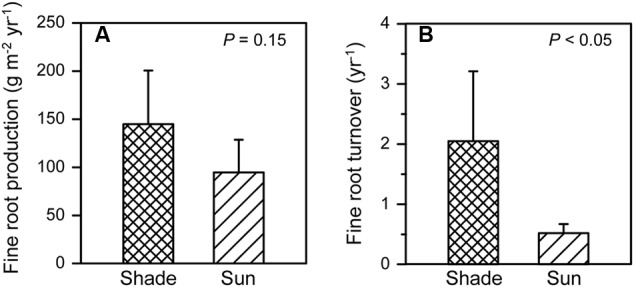
**Annual fine root production (A)** and turnover **(B)** in 2012 in the shaded area (i.e., upslope of the stem in a northerly direction) and the sunny area (ca. downslope of the stem in a southerly direction) in vicinity of the isolated 12 *P. cembra* trees (given are means + SE) in the vicinity of the six study plots in the year 2012.

Annual fine root growth into the ingrowth cores that were placed in vicinity of the 12 isolated *P. cembra* trees outside of the grid-sampling plots, was ∼50% higher in the shaded areas under the tree canopies than in the sunny areas, but this difference was only significant at a marginal probability level (**Figure 5A**). An even larger and significant difference was found for fine root turnover: in shaded patches, mean turnover was 2.1 year^-1^ while it was 0.5 year^-1^ in the sunny areas (*p* < 0.05) (**Figure 5B**). These figures are equivalent to a mean fine root lifespan <0.5 year in the shaded areas and of ∼2 year in the sunny areas.

## Discussion

The results support our first hypothesis on the biological significance of the afternoon temperature regime, but they do not support the second and third hypothesis concerning the temperature influence on fine root biomass and productivity. The size of the soil temperature differences between shaded and sunny patches in the plots is displayed by the temperature maps produced on August 1, and it is also evident from the long-term soil temperature measurements in July/August 2012 which show a nearly 2 Kelvin warmer soil in mid-summer in sunny plot areas. As postulated, the soil temperature regime recorded at dawn in the differently illuminated patches around the *P. cembra* trees did not explain the observed variation in fine root density or fine root length and surface area per soil volume in the six plots. The explained proportion of root biomass variation increased over the day with increasing heating of the sun-exposed areas in the plots, i.e., with growing soil temperature differences between shaded and sunny areas. Variation in fine root biomass density and fine root length and surface area per soil volume between the grid points is thus best explained by the afternoon temperature measurements. One might argue that the cold/shaded and warm/sunny zones were permanent or at least lasted for a significant period of time of the year. However, one should consider that at least for the shaded northern locations, in fact these places will be never sun-exposed during the whole year as there will be no direct sun irradiance from north at all. For the sunny, southern parts of the soil under the trees, it is indeed true that there is a certain change in the sun position during the year. However, one should take into account that the growing season at the alpine treeline in the Alps is only 4(-5) months. At least during this period, the southern places will definitely be unshaded.

More surprising is the finding that fine root biomass (and fine root length and surface area per soil volume) was not higher, but significantly lower, in plot areas with higher soil temperatures as compared to colder soil patches. In fact, the highest standing fine root biomass was found in the coldest, most shaded areas under the trees. These temperature differences also seem to affect root morphology. While most examined root traits and the degree of ectomycorrhizal colonization did not vary across locations differing in soil temperature, we found a significantly higher fine root tip frequency on the roots of colder patches: fine root strands from the 25% coldest sampling points had more than two times higher root tip frequencies than roots from the 25% warmest soil locations. It appears that *Pinus* roots in particularly cold soil zones tend to increase their nutrient uptake capacity by forming more root tips which are thought to be the root segments with highest uptake activity. Equally important is our result that fine root productivity as estimated by the ingrowth core experiment was stimulated by colder temperatures in summer. Cores installed in the shaded, colder patches north of the pine trees showed by 50% higher fine root growth than cores placed in more sunny areas south of the stems, contradicting our third hypothesis. This finding is important with respect to the assumption that self-shading is reducing tree productivity at the alpine treeline.

These results clearly point to a higher carbon allocation of *Pinus* to the fine root system in shaded, colder soil areas than in less shaded, warmer patches. This finding contrasts with the assumption that colder temperatures under the crown of single trees or under the canopy of closed stands in the treeline ecotone would impair root growth activity and result in lower fine root biomass, as it has been concluded from *ex situ* experiments with tree saplings (Turner and Streule, 1983; Häsler et al., 1999; Alvarez-Uria and Körner, 2007; Hoch and Körner, 2009; Schenker et al., 2014). Our findings are matching earlier observations that stand-level fine root biomass is often high at the alpine treeline compared to lower elevation stands (e.g., Helmisaari et al., 2007; Leuschner et al., 2007; Hertel and Wesche, 2008; Hertel et al., 2008; Hertel and Schöling, 2011a). Our results further confirm reports that fine root turnover is relatively high at the low soil temperatures which prevail at the alpine treeline (Graefe et al., 2008; Hertel and Schöling, 2011b; Moser et al., 2011; Mao et al., 2013). As our results were obtained from isolated trees, it is confirmed that the relatively high root biomass in the cold soil zones most likely is caused by the low soil temperatures or a factor related to them rather than being a consequence of high stem densities which typically increase in mountain forests toward the alpine treeline (e.g., Hertel and Wesche, 2008).

While it is well established that herbaceous plants increase their root:shoot biomass ratio with increasing elevation toward the alpine treeline (e.g., Körner and Renhardt, 1987), elevational patterns in belowground/aboveground biomass partitioning are barely understood in adult trees (Körner, 2012a). Our findings of a higher fine root biomass in shaded, colder patches at the treeline would be plausible, if trees were responding in a similar manner to reduced temperatures at higher elevations as herbs. To close the knowledge gap on C partitioning patterns of mountain forest trees, we compiled our own, mostly unpublished data from four elevation transect studies on the fine root biomass of mature trees in temperate and subtropical mountains (**Table 6**). Apart from the well-known phenomenon that tree density generally increases toward the alpine treeline, while tree height and aboveground tree biomass decrease, comparison of montane and treeline forests shows that the amount of fine root biomass per tree either remains constant (Mt. Brocken) or slightly decreases with elevation (Mt. Ventoux and Patagonian sites), reflecting the marked decrease in aboveground tree biomass with elevation. Physiologically more important is the result that the fine root-to-aboveground biomass ratio is much higher at the alpine treeline than in the montane forests. In the four transects, this ratio was 2- to 2.7-fold (Mt. Brocken, Mt. Ventoux, Mt. Tronador) or even 11 times higher at the treeline (El Chalten). The fine root-to-aboveground biomass ratios measured are also impressive in absolute terms: While fine root biomass typically represents only a few percent of total tree biomass in mature trees (e.g., Vogt et al., 1996; Finér et al., 2011b), this component accounts for about 10–15% of total biomass in the treeline stands of **Table 6** (an exception is Mt. Ventoux with only ca. 1%). These results confirm that trees at the treeline in temperate and subtropical mountains invest heavily in the root system, which is difficult to explain by the carbon sink limitation hypothesis.

**Table 6:** Compilation of fine root biomass data from four mountain ranges in Central Germany (Mt. Brocken), southern France (Mt. Ventoux) and two sites in the Patagonian (Argentinian) Andes (El Chalten, Mt. Tronador) comparing montane and treeline forests.

Site	Elevation (m asl.)	Mean annual stand air temperature (°C)	Species	Tree density (no. ha^-^^1^)	Mean tree height (m)	AGB (kg tree^-1^)	FRB (kg tree^-1^)	FRB/AGB (kg kg^-1^)
**Mt. Brocken (Germany, 51.5°N)**								
Montane forest	990	2.9	*Picea abies*	888	11.7	49.0	3.18	0.065
Treeline forest	1100	2.1	*Picea abies*	1406	5.9	17.4	3.06	0.177
**Mt. Ventoux (France, 44°N)**								
Montane forest	1510	n.a.	*Fagus sylvatica*	1800	10.9	226.6	1.34	0.006
Treeline forest	1760	n.a.	*Pinus uncinata*	3911	3.3	25.2	0.31	0.012
**Patagonian Andes (Argentina)**								
**El Chalten (49.3°S)**								
Montane forest	920	3.9	*Nothofagus pumilio*	800	25.8	297.5	3.06	0.010
Treeline forest	1050	2.8	*Nothofagus pumilio*	5625	3.6	9.9	1.06	0.107
**Mt. Tronador (41°S)**								
Montane forest	1410	4.9	*Nothofagus pumilio*	600	15.1	81.5	6.26	0.077
Treeline forest	1690	4.0	*Nothofagus pumilio*	8800	3.9	4.3	0.68	0.158

*Given are the elevation of the sites, mean annual air temperature inside the stands, the tree species building the stand, mean tree height, tree density, mean aboveground biomass (AGB) per tree, mean fine root biomass (FRB) per tree, and the FRB:AGB ratio. AGB data are based on calculations using allometric equations from the literature for the respective species or (if not available) for a related species with similar structural characteristics. Fine root biomass data were obtained from fine root inventory campaigns similar to that conducted in this study. Most root data are unpublished so far except for part of the data from Mt. Brocken (Hertel and Schöling, 2011a,b).*

The published information on the temperature dependence of tree root growth activity and the buildup of fine root biomass at treeline sites contains partly conflicting evidence. On the one hand, *ex situ* experiments with tree saplings or young trees in laboratory or garden environments in most cases revealed a linear (or non-linear) decrease of root growth rate with decreasing soil temperature, or they indicated low-temperature thresholds of root growth between 2 and ∼7°C (Bilan, 1967; Lopushinsky and Max, 1990; Häsler et al., 1999; Lahti et al., 2005; Alvarez-Uria and Körner, 2007; Hoch and Körner, 2009; Schenker et al., 2014). On the other hand, field studies of adult trees and forest stands indicated much more dynamic fine root growth in cold soils near the treeline than would be expected from the seedling or sapling experiments mentioned above. For example, Benecke et al. (1978) found that *Pinus contorta* and *Nothofagus solandri* at the treeline in New Zealand maintained notable fine root growth activity for at least 9 months of the year, although the conventionally defined growing season is much shorter. For *Picea abies* near the treeline in the European Alps, Sandhage-Hofmann and Zech (1993) observed large seasonal changes in fine root biomass and necromass (2.5-fold variation during 1.2 months) indicating remarkable fine root dynamics despite unfavorable cold growing conditions. Similarly, Hertel and Schöling (2011b) found in ingrowth cores a relatively high fine root productivity in a Norway spruce stand at the alpine treeline on Mt. Brocken (Central Germany) with a growing season soil temperature of 6.7°C and a mean annual soil temperature of 3.8°C. Based on direct observation in rhizoscopes, Mao et al. (2013) reported notable fine root elongation of *Abies alba* and *Picea abies* trees near the treeline in the French Alps at very cold soil temperature conditions. Using a minirhizotron approach, Sullivan et al. (2015) recorded fine root growth activity of adult *Picea glauca* trees even at soil temperatures of ca. 2.0°C; these authors found no difference in annual fine root productivity between two treeline forest stands in Alaska with mean growing season soil temperatures of 8.9 and 4.9°C. Using minirhizotrons, Gaul et al. (2008b) observed fine root growth in mature *Picea abies* trees in southern Germany at soil temperatures around the freezing point.

By comparing the temperature response of root growth of tree seedlings with the root dynamics of mature trees of the same species in cold soils in Alaska, Tryon and Chapin (1983) recognized a principle disagreement between *ex situ* and field data, as it was recognized above. In that study, mature trees in the field showed notable fine root growth activity even under very cold soil temperatures (<5°C), while sapling root growth was negatively influenced by low soil temperatures. Ruess et al. (2006) concluded from a review of fine root dynamics data from boreal forests that the fine roots of boreal conifers must have specific adaptations to function at low soil temperatures.

Greenhouse experiments with potted tree seedlings or saplings and field studies on mature trees may lead to different results because the soil constraints for root growth (soil volume, mycorrhization, root competition intensity) and the biological controls of belowground C allocation in the plants (carbohydrate storage, hormonal regulation of growth) may differ significantly. Moreover, the root growth of potted tree seedlings or saplings is generally explorative during the first months or even years of the experiment. Most of the fine roots of the young trees are built to access new soil volume and therefore increase the root biomass of the young tree, while root mortality is generally low in these early stages of a pot experiment (pers. observ., see also Aspelmeier, 2001; Beyer et al., 2013; Hajek et al., 2014). At field sites with mature long-established trees, the upper soil layers commonly are completely occupied by the fine root system of the trees, as long as no larger canopy gaps are present. Exploratory fine root growth at such sites normally is only of minor importance, while the major trigger for root growth activity is compensatory replacement of fine roots died. The magnitude of root mortality is thus an important factor influencing the growth activity of fine roots in established forests, and fine root growth and turnover will largely depend on variables affecting the lifespan of fine roots (Eissenstat and Yanai, 1997; Leuschner et al., 2001; Hertel and Leuschner, 2002; Gaul et al., 2008a; Hertel et al., 2013). This is also visible from the results of our study, where not only fine root biomass, but also fine root necromass was markedly higher in shaded, colder patches than in the sunny, warmer areas. Our finding that not only necromass but also fine root turnover was much higher in the colder soil patches in the shade compared to warmer areas (mean fine root longevity: ca. 0.5 vs. 2.0 years), confirms that the elevated fine root necromass values are not primarily a consequence of slower root decomposition due to hampered microbial activity, but rather are the result of higher fine root mortality at low temperatures. Similar results were obtained by Ruess et al. (2003) in a cold boreal forest site, where mature *Picea mariana* trees showed a high fine root turnover of 3.4 year^-1^ (i.e., a lifespan of only 108 days) despite a growing season soil temperature of only 3.0–5.6°C. In an experiment with artificial frost application, Gaul et al. (2008b) observed that fine root mortality due to winter frost stimulated compensatory fine root growth even under soil temperatures around the freezing point. Evidence for a stimulating effect of root mortality in cold soils to promote compensatory fine root growth is also presented in the studies of Ruess et al. (1998, 2003) and Weih and Karlsson (2002). Interestingly, our results clearly demonstrate that a stimulation of compensatory root growth (and hence a modification in belowground carbon allocation patterns) must result from autonomous stress sensing and signaling in individual fine root strands and not from a response of the whole tree individual. This is shown by the different responses of shaded colder and sun-exposed warmer root system components, which are part of the same *P. cembra* individual.

While the evidence for a negative relation between root zone temperature, and root biomass and root turnover at the studied treeline is striking, attempts to explain this phenomenon must remain speculative. According to the optimal resource partitioning theory, enhanced carbon allocation to the fine root system should indicate low availability of an essential soil resource (Bloom et al., 1985; Poorter and Nagel, 2000; Reich, 2002). This assumption would also explain why fine root mortality is higher under more stressful colder than under warmer soil conditions leading to higher fine root turnover. It is unlikely that soil water is limited at this site with >600 mm growing season precipitation although it cannot be principally excluded that differences in irradiance can also lead to temporary lack in soil water availability. We do not have direct measurements of soil water relations from the investigated soil patches in our plots (this would have required a separate study not only on differences in soil water content, but also investigations on surface runoff and tree water consumption). However, there is indirect evidence that soil moisture gradients are not very influential at the study plots. The ground vegetation (herbs and shrubs) did not differ between the different areas below the tree canopy and therefore indicate that the uniform slope angle of the site should have led to a relatively homogenous soil water content of the whole site. Accordingly, we speculate that one or more nutrient elements may be short in supply at the treeline plots. The temperature dependence of the processes controlling nutrient supply, including the activity of mineralizing microorganisms, nutrient diffusion in the soil, and the uptake kinetics of carriers in the root membranes, can result in reduced nutrient availability to plants in cold soils (e.g., Meentemeyer, 1977; Sveinbjörnsson et al., 1995; Timoney, 1995; Sveinbjörnsson, 2000; Müller et al., 2016). For example, the tree size and productivity decrease of *Picea abies* from submontane elevation to the alpine treeline on Mt. Brocken (Germany) was related not only to the temperature decrease but also to a reduction in net nitrogen mineralization rate (Plapp et al., unpublished data). In tropical montane forests in Ecuador, the aboveground productivity decrease toward the alpine treeline was associated with a fine root biomass and productivity increase, and decreases in soil pH, decomposition rate, and mineral N supply (Moser et al., 2011).

Trees in the treeline ecotone could compensate for reduced nutrient supply by increasing their absorbing fine root surface area and producing higher fine root tip frequencies at the cost of aboveground productivity. Such compensation should increase fitness particularly in the shaded colder patches of the ecotone. Soil acidity and low N availability may also reduce fine root longevity (e.g., Eissenstat and Yanai, 1997; Eissenstat et al., 2000) and thus could be among the causes leading to increased fine root mortality, as was observed in the colder patches of our study site. According to Sullivan et al. (2015), low nutrient availability is among the factors causing the alpine treeline in Alaska by mediating the effects of low temperature on above- and belowground productivity. This contradicts the statement of Körner (2003, 2012a,b) that nutrient deficiency should never dominate over unfavorable thermal growth conditions at the alpine treeline. Furthermore, this assumption (Körner, 2003, 2012a,b) is not consistent with the reports of a marked C allocation shift toward the root system in trees near the treeline. Low soil temperatures are also likely to impair root water uptake through a higher viscosity of water and reduced aquaporine-mediated water transport into the root xylem, but this has not been studied so far.

## Conclusion

The results of this systematic study on the temperature dependence of fine root biomass and fine root turnover in differently illuminated areas under the crown of isolated *P. cembra* trees in the treeline ecotone clearly show that self-shading does not impair fine root growth activity and the development of a large fine root system, despite lower temperatures in the shade. Rather, we found a higher root biomass density, higher root growth activity, and accelerated root turnover in the shade, which can only be interpreted as a compensatory response of the tree to reduced soil resource availability in a colder soil. Neither the carbon-source limitation hypothesis nor the carbon sink limitation hypothesis, which propose explanations for the halting of tree growth at the alpine treeline, can convincingly explain this phenomenon. This suggests that future efforts to achieve a causal explanation of the alpine treeline should explicitly consider plant-internal C allocation shifts and their possible abiotic causes. The role of nutrient deficiency as a possible factor contributing to alpine and arctic treelines deserves further study in this context. Our results on tree root biomass and dynamics at the treeline confirm the well-recognized fact that results from *ex situ* experiments with tree seedlings and saplings can rarely be transferred to the field. This calls for well-designed field studies on the C economy of trees below and at the treeline, which must include root dynamics.

## Author Contributions

DH, HC, and CL conceived the ideas and designed methodology; AG served for the grant and provided the infrastructure; PK, DH, AG and HC collected the data; PK and DH analyzed the data; PK, DH, CL and AG led the writing of the manuscript. All authors contributed critically to the drafts and gave final approval for publication.

## Conflict of Interest Statement

The authors declare that the research was conducted in the absence of any commercial or financial relationships that could be construed as a potential conflict of interest.
