# Supplementary material for: Fine Root Abundance and Dynamics of Stone Pine (Pinus cembra) at the Alpine Treeline Is Not Impaired by Self-shading
Source: Front Plant Sci. 2017 Apr 19;8:602. doi: 10.3389/fpls.2017.00602 (PMC5395556; doi:10.3389/fpls.2017.00602)
Supplement: Supplementary file 1 [file Image_1.PDF]

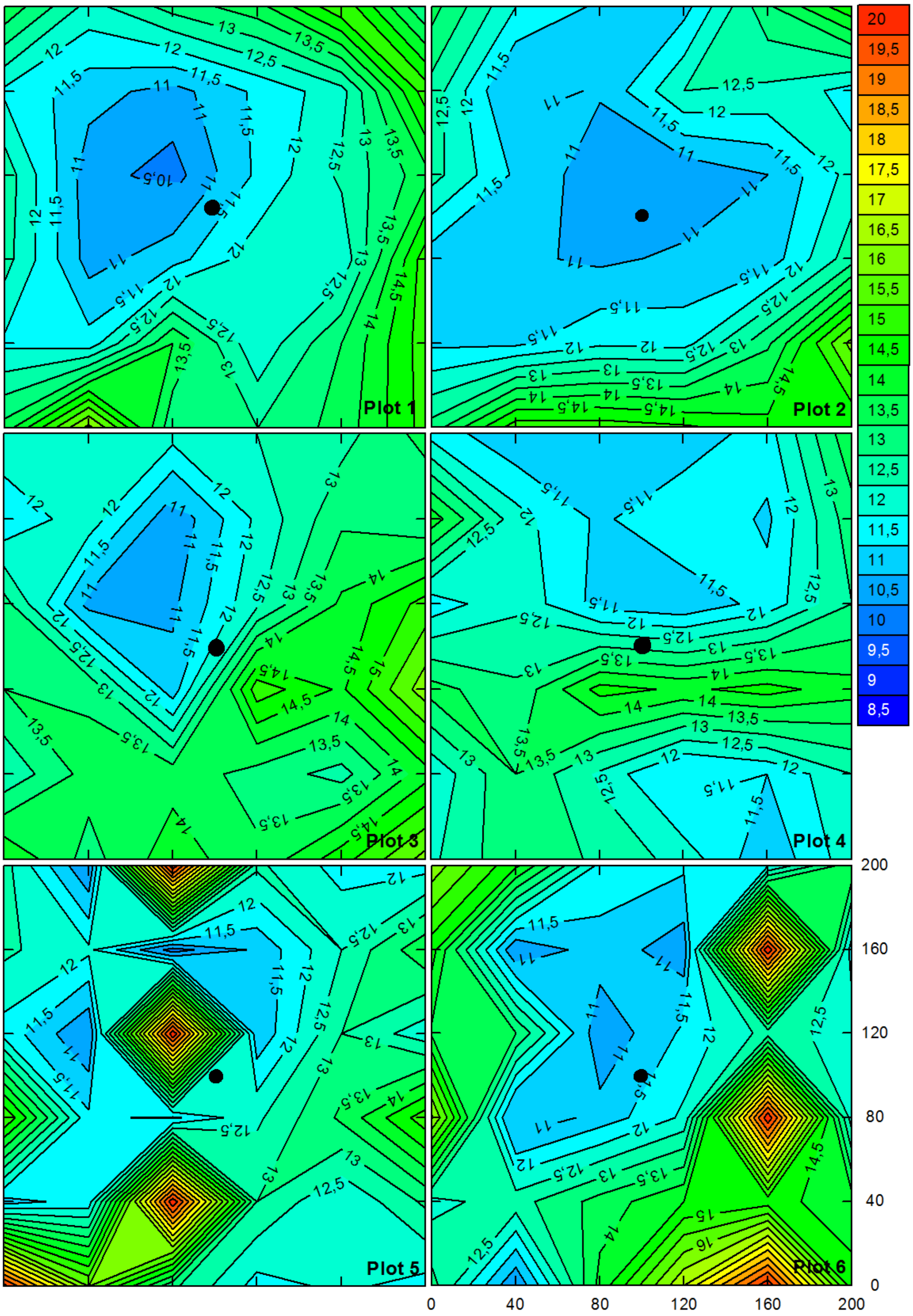

Figure SI1. Soil temperature isotherms in the six 4 m^2-plots around the *Pinus cembra* trees, interpolated from 36 temperature measurements per plot at 10 cm depth in the afternoon period. Black dots mark the position of the tree stem in the plots. All graphs are oriented to north.
